# Polarity and cell division orientation in the cleavage embryo: from worm to human

**DOI:** 10.1093/molehr/gav068

**Published:** 2016-10-05

**Authors:** Anna Ajduk, Magdalena Zernicka-Goetz

**Affiliations:** 1 Department of Embryology, Faculty of Biology, University of Warsaw, Miecznikowa 1, 02-096 Warsaw, Poland; 2 Department of Physiology, Development and Neuroscience, University of Cambridge, Downing Street, Cambridge CB2 3DY, UK

**Keywords:** polarity, par proteins, Hippo signalling, cytoplasmic flow, embryo, *C. elegans*, *Drosophila*, mouse, human, preimplantation development

## Abstract

Cleavage is a period after fertilization, when a 1-cell embryo starts developing into a multicellular organism. Due to a series of mitotic divisions, the large volume of a fertilized egg is divided into numerous smaller, nucleated cells—blastomeres. Embryos of different phyla divide according to different patterns, but molecular mechanism of these early divisions remains surprisingly conserved. In the present paper, we describe how polarity cues, cytoskeleton and cell-to-cell communication interact with each other to regulate orientation of the early embryonic division planes in model animals such as *Caenorhabditis elegans*, *Drosophila* and mouse. We focus particularly on the Par pathway and the actin-driven cytoplasmic flows that accompany it. We also describe a unique interplay between Par proteins and the Hippo pathway in cleavage mammalian embryos. Moreover, we discuss the potential meaning of polarity, cytoplasmic dynamics and cell-to-cell communication as quality biomarkers of human embryos.

## Introduction

Cleavage is a period after fertilization, when a 1-cell embryo starts developing into a multicellular organism. It consists of a series of mitotic divisions, which divide the large volume of a fertilized egg into numerous smaller, nucleated cells—blastomeres.

The pattern of cleavage divisions differs between species. The main factor determining where cleavage can occur, regulating size of the blastomeres and division timings is yolk. In general, blastomeres formed in the relatively yolk-free animal pole are smaller than those on the vegetal, yolk-rich pole. A good example is an amphibian egg, with a moderate vegetal yolk deposition (mesolecithal egg). During cleavage, small blastomeres (micromeres) are formed in the animal pole, whereas big blastomeres (macromeres)—in the vegetal pole. However, as amount of yolk differs between species, the cleavage pattern observed in amphibians is not universal. Mammalian eggs have no yolk (alecithal eggs) and blastomeres created during cleavage are of equal size. At the other extreme are eggs of insects, fish, reptile and birds. Most of their volume is filled with yolk and they undergo a meroblastic (partial cleavage) with cleavage furrows penetrating only a portion of the cytoplasm. In insect (e.g. *Drosophila*) eggs, yolk is localized in the cell centre and cleavage occurs in the cortical area (i.e. superficial cleavage). Conversely, in fish, reptiles and birds, the cleavage divisions take place only in a small disc of cytoplasm at the animal pole of the egg (discoidal cleavage) (reviewed in Gilbert, 2013).

In spite of this diversity in cleavage patterns, molecular mechanisms regulating early embryonic divisions remain strongly conserved among phyla. Interactions between polarity cues, cytoskeleton and cell-to-cell communication are crucial for cleavage in a wide range of examined species, from *Caenorhabditis elegans* to mouse. In the present paper, we wish to present these universal mechanisms that together guide embryos through the cleavage and discuss their potential meaning as quality biomarkers of human embryos.

## Polarity and spindle orientation

**Figure 1:**
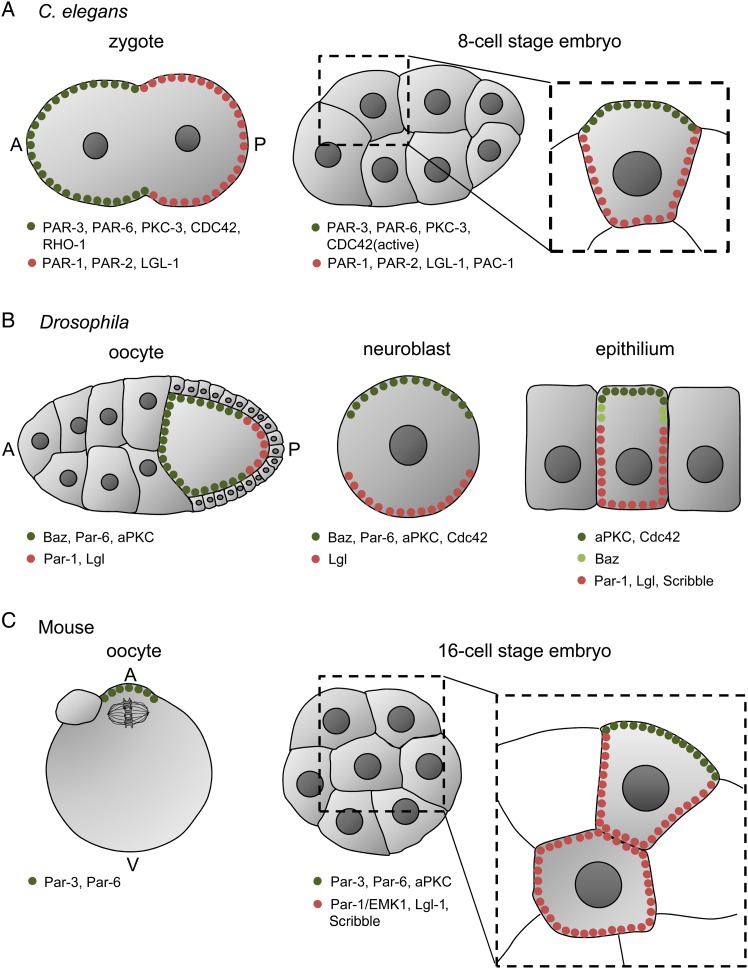
Polarity in *C. elegans*, *Drosophila* and mouse oocytes and embryos. Polarized distribution of PAR proteins and accompanying factors in *C. elegans* zygote and 8-cell stage embryo (**A**), *Drosophila* oocyte, neuroblast and epithelium (**B**), and mouse oocyte and 16-cell stage embryo (**C**). In (A) and (B): A, anterior pole; P, posterior pole. In (C): A, animal pole; V, vegetal pole.

Establishment of cellular polarity is one of the most important events during early embryonic divisions. In most species, including mammals, it enables cells to adopt distinct developmental fates. The main signalling pathway involved in cell polarization, mediated by the PAR (partitioning defective) proteins, was discovered in *C. elegans* embryos due to its ability to affect asymmetry of the first cleavage division (Kemphues *et al*., 1988). Before fertilization, PAR-3, PAR-6 and PKC-3 (the nematode homologue of aPKC) are present throughout the egg cortex and PAR-1 and PAR-2 localize in the cytoplasm (Munro and Bowerman, 2009; Nance and Zallen, 2011). After fertilization, PAR proteins and PKC-3 become redistributed in a polarized manner with PAR-1 and 2 found in the cortex above the sperm-derived centrosome (marking it as the posterior pole) (Guo and Kemphues, 1995; Boyd *et al*., 1996), and PAR-3, PAR-6 and PKC-3 located to the cortex of the opposite, anterior pole (Fig. 1A) (Etemad-Moghadam *et al*., 1995; Tabuse *et al*., 1998; Hung and Kemphues, 1999). PAR-4 and PAR-5 in contrast are localized uniformly in the whole cortex of the 1-cell embryo (Watts *et al*., 2000; Morton *et al*., 2002). When polarity is already established, Rho GTPases such as CDC42 and RHO1 (homologue of vertebrate RhoA) also become enriched in the anterior pole, participating in polarity maintenance (Aceto *et al*., 2006; Motegi and Sugimoto, 2006; Schonegg and Hyman, 2006). On the other hand, the posterior pole accumulates the protein LGL-1, reported to act redundantly with PAR-2 to maintain PAR asymmetry (Beatty *et al*., 2010, 2013; Hoege *et al*., 2010).

**Figure 2:**
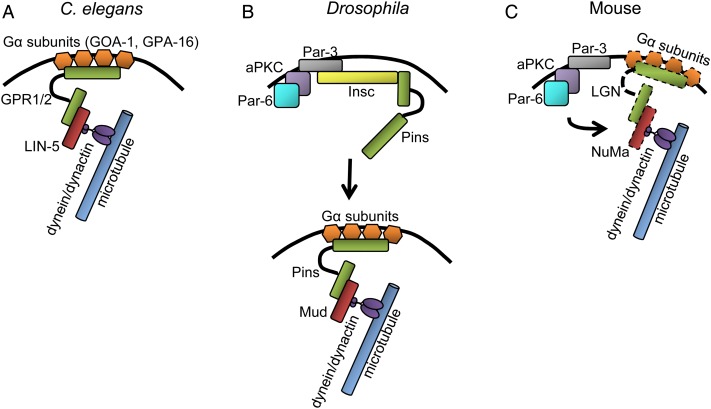
Interplay between PAR proteins and microtubules. Mechanism of a polarized dynein/dynactin-driven force exerted on microtubules in *C. elegans* (**A**), *Drosophila* (**B**) and mouse (**C**). Details in the main text. Boxes encircled with a dashed-line symbolize proteins that were not directly confirmed in the PAR proteins–microtubule interactions in the cleavage mouse embryos.

During the first division, the interaction between astral microtubules emanating from the spindle poles and the cortex leads to an asymmetric localization of the spindle, as it is pulled towards the posterior part of the zygote. Due to this dislocation, the first cleavage division results in two daughter cells of unequal size, termed the AB and P1 blastomeres (Nance and Zallen, 2011; Rose and Gönczy, 2014). These cells differ not only in size, but also inherit different complements of cell fate determinants, enabling them to follow distinct developmental paths. It seems that translocation of the spindle depends on a ternary protein complex comprised of two partially redundant protein Gα subunits GOA-1 and GPA-16, two essentially identical proteins containing GoLoco domain, GPR1 and GPR2, and the protein LIN-5 (Fig. 2A) (Gotta and Ahringer, 2001; Colombo *et al*., 2003; Gotta *et al*., 2003; Srinivasan *et al*., 2003; Tsou *et al*., 2003). As Gα proteins are myristoylated, the entire complex is anchored in the cell membrane. It is still unclear how the asymmetric pulling force is generated by the complex. However, it is probable that it may be related to the slightly asymmetric distribution of the complex components: whereby GOA-1 and GPA-16 are distributed uniformly throughout the cell cortex and LIN-5 is localized equally at both cell poles, GPR1 and 2 are enriched in the posterior region (Miller and Rand, 2000; Gotta and Ahringer, 2001; Colombo *et al*., 2003; Gotta *et al*., 2003; Tsou *et al*., 2003; Afshar *et al*., 2004, 2005; Park and Rose, 2008). Moreover, asymmetry in GPR1 and 2 localization depends on anterior–posterior polarization (Gotta and Ahringer, 2001; Colombo *et al*., 2003; Gotta *et al*., 2003; Srinivasan *et al*., 2003; Tsou *et al*., 2003), suggesting that it can be indeed essential for the asymmetry in the pulling forces and translocation of the mitotic spindle. LIN-5 is a homologue of NuMa that can associate in vertebrate cells with dynein, a microtubule motor protein (Merdes *et al*., 1996; Kotak *et al*., 2012). Therefore, it is thought that the ternary complex binds through LIN-5 to a dynein/dynactin complex and in consequence can generate pulling force on spindle astral microtubules (Fig. 2A) (Rose and Gönczy, 2014). There is also a possibility that posteriorly directed pulling force is, at least partially, caused by anchoring and stabilization of anterior microtubules by PAR-3 (Labbé *et al*., 2003). PAR-2 and PAR-3 are known to regulate pulling forces exerted on both sides of the spindle during spindle positioning (Grill *et al*., 2001); however, as the microtubules are stabilized by anteriorly accumulated PAR-3, the net force is directed towards the more dynamic, posterior pole (Labbé *et al*., 2003).

At the 4-cell stage, the pattern of PAR asymmetry changes, as the *C. elegans* embryo becomes polarized radially. Blastomeres at this stage are named ABa and ABp (derived from AB blastomere) and EMS and P2 (derived from P1 blastomere). Interestingly, in contrast to other model organisms, radial polarization of *C. elegans* blastomeres does not affect cell fate but rather influences events during early gastrulation (Nance *et al*., 2003). PAR-3, PAR-6 and PKC-3 are absent at the cell contact sites and accumulate in the contact-free surfaces (Etemad-Moghadam *et al*., 1995; Hung and Kemphues, 1999; Nance and Priess, 2002; Nance *et al*., 2003). At the same time, PAR-1 and PAR-2 become enriched in the surfaces between blastomeres (Fig. 1A) (Nance and Priess, 2002; Nance *et al*., 2003). This process is probably mediated by PKC-3 that localizes in contact-free surfaces in a PAR-3 and PAR-6-dependent manner (Tabuse *et al*., 1998; Nance *et al*., 2003) and phosphorylates PAR-2, blocking its cortical binding (Hao *et al*., 2006). As PAR-1 depends on PAR-2 in its localization, it also cannot localize to the cell apical cortex (Motegi *et al*., 2011). The localization of the mitotic spindle under such conditions depends, at least partially, on proteins expressed also in the zygote stage. GPR1, GPR2 and LIN-5 regulate spindle position during the asymmetric division of P2 blastomere (Srinivasan *et al*., 2003; Tsou *et al*., 2003; Werts *et al*., 2011), whereas GPA-16 participates in spindle orientation in the asymmetric division of the EMS and symmetric divisions of the ABa and ABp blastomeres (Bergmann *et al*., 2003; Tsou *et al*., 2003; Zhang *et al*., 2008). Interestingly, in the EMS, blastomere spindle position is also regulated by selected components of the Src and Wnt signalling pathways (Schlesinger *et al*., 1999; Bei *et al*., 2002; Zhang *et al*., 2008; Werts *et al*., 2011; Rose and Gönczy, 2014).

Par proteins also participate in the establishment of polarity in *Drosophila* embryos, both in single cells and multicellular epithelia. The Par3 homologue, Bazooka (Baz), was discovered in fly embryos in the 1980s (Wieschaus *et al*., 1984), and later other Par-related components were isolated, including: Par-1, aPKC (homologue of PKC-3), Par-6, Lkb-1 (homologue of PAR-4) and 14-3-3ε and ζ (homologues of PAR-5) (Shulman *et al*., 2000; Tomancak *et al*., 2000; Wodarz *et al*., 2000; Petronczki and Knoblich, 2001; Benton *et al*., 2002; Martin and St Johnston, 2003). The anterior–posterior axis in *Drosophila* is formed before fertilization with Baz, Par-6 and aPKC localizing to the anterior and lateral cortex of the oocyte, and Par-1 and Lgl (homologue of *C. elegans* LGL-1) to the posterior cortex (Fig. 1B) (reviewed in Nance and Zallen, 2011). aPKC phosphorylates Par-1 and Lgl, thus excluding them from the anterior and lateral cortex and restricting their localization to the posterior region (Hurov *et al*., 2004; Tian and Deng, 2008; Doerflinger *et al*., 2010). Conversely, Par-1 phosphorylates Baz and excludes it from the posterior domain (Vaccari and Ephrussi, 2002; Benton and St Johnston, 2003).

The Par pathway also participates in polarity establishment during later stages of *Drosophila* embryonic development, such as in neuroblasts and epithelial cells. In neuroblasts, Baz, aPKC, Par-6 and Cdc42 form an apical domain that is limited, at least in part, by antagonism with basally located Lgl (Fig. 1B) (Prehoda, 2009; Bergstralh *et al*., 2013). In epithelial cells on the other hand, the apical domain is enriched with aPKC and Cdc42, whereas the basolateral cortex accumulates Par-1, Lgl, Dlg and Scribble. Baz is present in the adherens junction sites, at the boundary between apical and basolateral domains (Fig. 1B) (reviewed in Bergstralh *et al*., 2013). As in *Drosophila* oocytes, aPKC also phosphorylates Par-1 and Lgl in epithelial cells, causing their basolateral translocation, whereas Par-1 phosphorylates Baz excluding it from the basolateral cortex (Benton and St Johnston, 2003; Rolls *et al*., 2003; Betschinger *et al*., 2005; Nance and Zallen, 2011; Bergstralh *et al*., 2013).

The *Drosophila* neuroblast is an ideal model for studying the interplay between polarization, spindle orientation and asymmetric cell division (Fig. 2B). Neuroblasts express Inscuteable (Insc), that localizes apically due to its interaction with aPKC/Par-6/Baz (Schober *et al*., 1999; Wodarz *et al*., 1999). Insc is responsible for recruiting the GoLoco domain containing protein, Partner of Inscuteable (Pins, a homologue of vertebrate LGN) to the apical membrane. This interaction promotes spindle orientation along the apical–basal axis. Pins can bind to either Insc or Mud (a homologue of vertebrate NuMa and *C. elegans* LIN-5) but not to both simultaneously (Culurgioni *et al*., 2011; Yuzawa *et al*., 2011; Zhu *et al*., 2011), suggesting that Insc recruits Pins to the membrane, but then it has to pass it to Mud. This does not necessarily remove Pins from the apical cortex, as Pins may interact with cortical subunits Gα. Once bound to Mud, Pins can exert pulling forces on astral spindle microtubules through dynein/dynactin complex (Fig. 2B). Considerably less attention has been paid to spindle orientation in symmetrically dividing *Drosophila* epithelial cells; however, it seems that the interaction between aPKC and Pins also plays a role there (Bergstralh *et al*., 2013).

In mouse, polarity is first established in unfertilized oocytes characterized by the metaphase spindle localized to the animal hemisphere of the oocyte. Chromosomal proximity leads to cortex reorganization in a Ran GTPase-dependent manner and induces actin, myosin 2, Par-3 and Par-6 accumulation above the spindle (Fig. 1C) (Vinot *et al*., 2004; Duncan *et al*., 2005; Deng *et al*., 2007; Ajduk *et al*., 2013). Several studies suggest that Cdc42, Rac-GTPase and the Mos/MEK/MAP kinase pathway may be also involved in the reorganization of the cortex above the spindle, as they regulate the translocation of the meiotic spindle to the oocyte cortex (Araki *et al*., 1996; Choi *et al*., 1996; Verlhac *et al*., 2000; Tong *et al*., 2003; Deng *et al*., 2005; Na and Zernicka-Goetz, 2006; Halet and Carroll, 2007; Yu *et al*., 2007).

**Figure 3:**
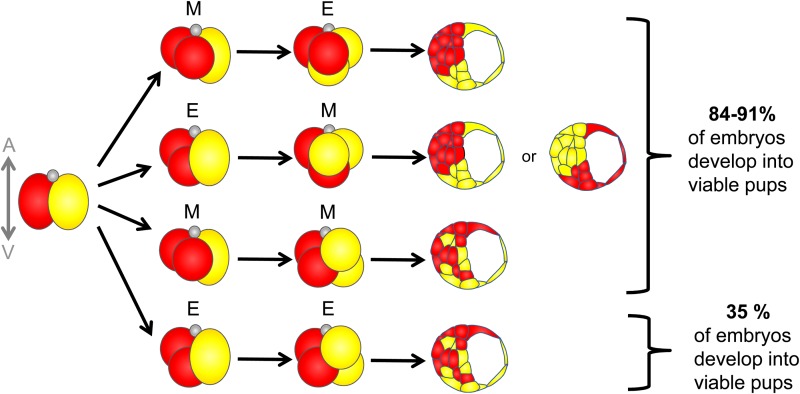
Cleavage patterns of mouse embryos at 2- to 4-cell transition. Two-cell stage mouse blastomeres divide either meridionally (M) or equatorially (E) giving rise to four types of embryos: ME, EM, MM and EE. Depending on the cleavage plane, progeny of the blastomeres populate different regions of the blastocyst (for details, see the main text). Moreover, embryos, which underwent at least one meridional division (ME, EM and MM), develop to term significantly more efficiently than EE embryos (data from Piotrowska-Nitsche and Zernicka-Goetz, 2005). A, animal pole; V, vegetal pole.

After fertilization, the clear asymmetry observed in oocytes either entirely or largely disappears. However, it is likely that some, unknown yet, components, localized in a polarized way along the animal–vegetal axis, still affect cell divisions and developmental potential of the blastomeres. The first cleavage plane in zygotes is almost exclusively meridional (M), which means it occurs along the animal–vegetal axis (Howlett and Bolton, 1985; Gardner, 1997; Plusa *et al*., 2002; Piotrowska-Nitsche and Zernicka-Goetz, 2005) resulting in an equal inheritance of cytoplasm from the animal and vegetal zygotic regions between both blastomeres in the 2-cell stage embryo. However, the divisions of the second cleavage can be orientated either meridionally, like in zygotes, or equatorially (E), i.e. perpendicular to the animal–vegetal axis with the animal and vegetal material inherited differentially by different daughter cells (Fig. 3) (Gardner, 2002; Piotrowska-Nitsche and Zernicka-Goetz, 2005). Therefore, and after considering the relative order of timing between the two asynchronous cell divisions, four distinct types of 4-cell stage embryo can be distinguished: ME, EM, MM and EE. It has been shown that the pattern of the second cleavage division, as well as the order in which these divisions occur, affects both fate and developmental potential of the resulting cells (Piotrowska-Nitsche and Zernicka-Goetz, 2005). In the majority of ME embryos, the embryonic part of the blastocyst (so-called inner cell mass, ICM) is built by the progeny of the 2-cell blastomere that divided meridionally. Moreover, the progeny of the E-blastomere that inherited the vegetal material subsequently undertake significantly more symmetric divisions compared with the progeny of other blastomeres (Bischoff *et al*., 2008), thus preferentially contribute to the mural trophectoderm (i.e. the abembryonic part of the blastocyst; Piotrowska-Nitsche *et al*., 2005). In comparison, progeny of the E-blastomere containing animal material predominantly populates the boundary zone between ICM and trophectoderm (Piotrowska-Nitsche *et al*., 2005). EM embryos, on the other hand, have embryonic and abembryonic regions in the blastocyst built mainly by progeny of one of the 2-cell stage blastomere, but this can be either a clone originating from equatorial or meridional division. In contrast, the relationship between division orientation and cell allocation in blastocyst appears completely random in MM and EE embryos (Fig. 3). Importantly, the pattern of segregation and inheritance of animal and vegetal material also correlates with developmental potential of embryos. The majority of embryos in which at least two 4-cell stage blastomeres inherit both animal and vegetal material (i.e. embryos that have at least one meridional division: ME, EM, MM), develop successfully to term, whereas only one-third of embryos in which all 4-cell stage blastomeres have exclusively either animal or vegetal material (EE embryos) give rise to viable pups (Fig. 3) (Piotrowska-Nitsche and Zernicka-Goetz, 2005; Piotrowska-Nitsche *et al*., 2005).

The exact molecular mechanism behind this cleavage-related asymmetry still remains to be discovered and is the subject of intensive research. Molecules distributed differentially between animal and vegetal part of the mammalian zygote that could be responsible for different cell fate of the blastomeres that inherited them are still unknown. The candidates proposed so far are: hormone leptin, transcription factor STAT3, growth factors TGFβ2 and VEGF, and the apoptosis-associated proteins BCL-X and BAX (Antczak and Van Blerkom, 1997, 1999; Schulz and Roberts, 2011). Unfortunately, apart from asymmetric localization of these proteins, there is no proof for a functional link between them and developmental fate of the blastomeres. It has been found however, that in ME embryos, cells inheriting more vegetal part of the zygote have lower levels of histone H3 R26/17 methylation when compared with the remaining cells at the 4-cell stage (Torres-Padilla *et al*., 2007). Moreover, it has been shown that blastomeres with high levels of Carm1 methylotransferase, an enzyme responsible for this specific H3 arginine methylation, up-regulate pluripotency markers, such as Nanog and Sox2 and their progeny tends to localize in the ICM (Torres-Padilla *et al*., 2007; Parfitt and Zernicka-Goetz, 2010). In support of these findings, another study revealed that 8-cell blastomeres originated from equatorial division in ME embryo are characterized by 5-fold higher expression of a trophectoderm marker, Cdx2 (Jedrusik *et al*., 2008). Furthermore, Plachta *et al*. (2011) have found that blastomeres at 4-cell and 8-cell stage differ in kinetics of nuclear import/export of another key pluripotency factor Oct4. Cells with a stable nuclear pool of Oct4 take more asymmetric divisions and preferentially develop to ICM, whereas cells with more ‘mobile’ nuclear Oct4 tend to divide symmetrically and differentiate into trophectoderm. Therefore, it is plausible that these differences in Oct4 kinetics originate from the animal–vegetal asymmetries formed initially within the oocyte.

Par-related polarity becomes re-established in mouse embryos at the 8-cell stage, but this time, as in *C. elegans*, the polarity is radial. During this process, mouse blastomeres increase intercellular adhesion (i.e. undergo compaction) and a clear distinction between apical and basolateral surfaces develops. Similarly to *C. elegans* and *Drosophila* cells, the apical region in mouse blastomeres is enriched in Par-6 and aPKC proteins, as well as in F-actin, whereas basolateral parts accumulate Par-1/EMK1. At later stages, the Par-3 protein joins Par-6 and aPKC in the apical domain (Fig. 1C) (Pauken and Capco, 2000; Plusa *et al*., 2005; Vinot *et al*., 2005). Other basolateral polarity regulators, such as Lgl-1 and Scribble, are also present in cell contact sites and they seem to be directed to this localization by activity of aPKC and Par6 (Hirate *et al*., 2013). During 8- to 16- and 16- to 32-cell transitions, a portion of cells divides at the angle that is more perpendicular than parallel to the embryo surface, producing outer polarized cells and inner apolar cells. Both of these cells display different fates, with inner cells forming ICM in the blastocyst stage, and outer cells—trophectoderm (reviewed in Zernicka-Goetz *et al*., 2009). The different fates of these cells in mammals are defined by differences in cell polarity and in the expression pattern of key transcription factors triggered by both cell position (i.e. inner or outer) and a distinct localization of transcripts [e.g. apically biased localization of Cdx2 mRNA, facilitating its inheritance by the outer cells (Jedrusik *et al*., 2008; Skamagki *et al*., 2013)].

A mechanism governing the orientation of division planes in a cleavage mouse embryo remains largely elusive; however, recent papers provide some important insights. It has been shown that division plane correlates in the 8-cell stage blastomere with the position of the nucleus. Blastomeres with nuclei located apically divide almost exclusively symmetrically, whereas blastomeres with nuclei situated in the basolateral region divide either symmetrically or asymmetrically (Ajduk *et al*., 2014). Apical localization of the nuclei is facilitated by aPKC activity, as overexpression of dominant negative version of aPKC leads to more nuclei relocated basally (Ajduk *et al*., 2014). Importantly, lack of or diminished aPKC activity also results in an increased frequency of asymmetric divisions or cell displacement from the surface to the inside of the embryo (Plusa *et al*., 2005; Ajduk *et al*., 2014) induced by constrictions of the apical actomyosin network (Samarage *et al*., 2015). Nucleus position seems to be regulated by kinesin and dynein microtubule motor proteins: basal displacement of blastomere nuclei depends on kinesins and is counteracted by dynein-driven forces, and is probably, analogous to the situation in *C. elegans* and *Drosophila* embryos. Moreover, aPKC also promotes movement towards the apical cortex (Ajduk *et al*., 2014). In other mammalian cell systems, aPKC and dynein/dynactin-driven force is exerted on microtubules via proteins such as Gα, LGN and NuMa (reviewed in Siller and Doe, 2009); therefore, it seems plausible that they too may participate during nuclear positioning in 8-cell stage blastomeres (Fig. 2C).

## Cytoplasmic flow and a polarity determinants distribution

Establishment of polarity in embryos requires displacement of various cell fate determinants. This redistribution is often mediated by a cytoplasmic flow, a directional cytoplasmic movement dependent on actomyosin cytoskeleton. The mechanism of cytoplasmic flow was examined in detail in *C. elegans* embryos. Some analogical processes have been also identified in mammalian oocytes and zygotes (Deguchi *et al*., 2000; Ajduk *et al*., 2011; Yi *et al*., 2011a, b), but there is currently no evidence for asymmetric actomyosin flow related to polarity in *Drosophila* oocytes or embryos. However, a microtubule- and kinesin-dependent cytoplasmic streaming has been identified in *Drosophila* oocytes. Its function is not completely clear, but the streaming seems to be involved in establishment of the polarity (Glotzer *et al*., 1997; Serbus *et al*., 2005; Ganguly *et al*., 2012).

In *C. elegans* egg, cytoplasmic flow is induced by fertilization. The egg is filled with a dynamic and contractile actomyosin network, which becomes destabilized at the sperm entry site. It initiates a flow of cortical non-muscle myosin 2 (NMY-2) and F-actin towards the opposite, anterior, pole. PAR-3, PAR-6 and PKC-3, as well as non-PAR proteins that associate with the cytoskeleton, appear to be transported to the anterior by this movement (Munro *et al*., 2004; Nance and Zallen, 2011). In turn, PAR proteins modulate actomyosin dynamics. PAR-3, PAR-6 and PKC-3 promote the cortical flow (Munro *et al*., 2004). PAR-2, which localizes to the posterior cortex, inhibits NMY-2 from accumulating there during the flow, and maintains asymmetry by preventing inappropriate, posterior-directed flows (Munro *et al*., 2004). PAR-4, on the other hand, facilitates actomyosin contractility and its depletion leads to reduced mobility of NMY-2, mislocalization of anterior PAR proteins and defects in cytokinesis (Chartier *et al*., 2011). It seems that PAR-4 affects actomyosin contractility, and in turn PAR asymmetric distribution, regulating activity of actin cytoskeleton scaffold protein anillin (Chartier *et al*., 2011). Myosin is also regulated by RHO1 small GTPase, which phosphorylates and activates MLC4, the myosin regulatory light chain subunit (Jenkins *et al*., 2006). RHO1 is activated by Rho guanine nucleotide exchange factors (RhoGEFs), with ECT-2 being the principal one (Jenkins *et al*., 2006; Motegi and Sugimoto, 2006; Schonegg and Hyman, 2006), and inhibited by Rho guanosine triphosphatase activating proteins (RhoGAPs), such as CYK-4, RGA3 and 4 (Jenkins *et al*., 2006; Schmutz *et al*., 2007; Schonegg *et al*., 2007).

In later cleavage stages of *C. elegans* embryo, cortical flow also plays an important role. A rotational cortical flow orthogonal to the anterior–posterior axis occurs during the division of the AB blastomere and positions the cytokinetic midbody remnant of the previous division asymmetrically at the future ventral side of the embryo, participating in establishment of the embryonic dorsoventral axis (Singh and Pohl, 2014).

Actomyosin-dependent cytoplasmic flow has also been described in mouse oocytes and embryos. In unfertilized oocytes, actin filaments flow continuously away from the animal cortex, inducing a cytoplasmic streaming exerting a net pushing force on the spindle towards the cortex. This flow is regulated by Arp2/3 complex, a nucleator of branched actin filaments, which accumulates to a cortical cap above the spindle in a Ran-GTPase-dependent way. Arp2/3 inhibition not only diminishes the flow but also enables a reverse streaming driven by myosin 2-based cortical contraction, moving the spindle away from the cortex (Yi *et al*., 2011a, b). Therefore, the asymmetric spindle position seems to be maintained by balanced forces governed by the Arp2/3 complex. The asymmetric localization of metaphase spindle is also regulated by Rac GTPase, as its inhibition leads to a displacement of the spindle from the cortex to the central region (Halet and Carroll, 2007).

Actomyosin-induced cytoplasmic streaming has also been identified in mouse oocytes upon fertilization (Deguchi *et al*., 2000; Ajduk *et al*., 2011). Sperm entry drastically changes the dynamics of cytoplasmic movements, leading to rhythmical cytoplasmic flows. They are caused by contractions of the actomyosin cytoskeleton triggered by Ca^2+^ oscillations induced by the sperm. The whole actomyosin cytoskeleton in the zygote contracts during the Ca^2+^ transients, but, due to its asymmetric distribution (a strong enrichment in the cortical caps above the oocyte and sperm chromosomes), the cytoplasmic movement becomes directional. It has two phases that can be easily distinguished during each Ca^2+^ transient: first, the cytoplasm moves towards cortical actomyosin caps, and then it retracts (Ajduk *et al*., 2011). Although similar Ca^2+^- and actomyosin-dependent cytoplasmic flows occur in ascidians and sea urchins and have been shown to play an important role in the establishment of embryo polarity (Speksnijder *et al*., 1990; Roegiers *et al*., 1995; Sardet *et al*., 2007), their function in mammalian zygotes remains elusive. Actomyosin-driven contractile waves have been also described in 8-cell stage mouse embryo upon compaction. The contractions occur periodically and are regulated by E-cadherin that appears to redirect contractility away from the cell–cell contacts (Maitre *et al*., 2015). Moreover, a recent study suggests that cortical tension generated by actomyosin contractility is the main factor responsible for internalization of a subset of cells (precursors of the ICM) at 8- to 16-cell stage transition (Samarage *et al*., 2015).

## Cell-to-cell communication as a polarity cue

In many biological systems, polarization events depend on intracellular contacts. Similar relationships can be observed during embryonic development, although their extent differs between species. In *C. elegans* embryos, cell contacts are necessary for establishing and maintaining polarity, while in mammals (mice)—only for its establishment. In some species, e.g. *Xenopus*, connections between blastomeres are neither required for setting up nor maintaining cellular polarity (reviewed in Nance, 2014).

In *C. elegans* embryo, cell-to-cell communication is necessary in radial polarization of somatic blastomeres at the 4-cell stage (Nance, 2014). During early cleavage divisions, cells do not form cell junctions but remain adherent and can receive signals from cell contact sites. E-cadherin HMR-1 together with α-catenin HMP-1, p120 catenin JAC-1, and linker protein PICC-1 recruit the RhoGAP protein PAC-1 (homologue of vertebrate Arghgap 21), a CDC42 inhibitor, to the cell contact sites (Anderson *et al*., 2008; Klompstra *et al*., 2015). CDC42 is distributed uniformly in the blastomere cortex, but due to PAC-1 polarized localization, it becomes inactivated in the cell contact surfaces. On the other hand, its activity in the apical cortex is sustained by RhoGEFs such as CGEF-1 and ECT-2 (Kumfer *et al*., 2010; Chan and Nance, 2013). As only active CDC42 can recruit PAR-6, the localization of PAR-6 and as a consequence PAR-3 and PKC-3 becomes restricted to the apical, cell contact-free, blastomere domain, leaving basolateral cortex enriched only for PAR-1 and -2 proteins (Gotta *et al*., 2001; Nance *et al*., 2003; Aceto *et al*., 2006; Anderson *et al*., 2008; Nance, 2014).

In *Drosophila* embryos, the interplay between cell-to-cell communication and polarity has been best examined in the epithelium. Par proteins collaborate with adherens junction proteins (e.g. E-cadherin, α- and β-catenin) to generate the apical–basal polarity. Adherens junctions mark the boundary between apical and basolateral domains and mediate interactions between cells (Nishimura and Takeichi, 2009; Nance and Zallen, 2011). Lack of Baz leads to mislocalization of the adherens junctions that fail to locate to the apicolateral membrane and become distributed throughout the basolateral domain (Müller and Wieschaus, 1996; Harris and Peifer, 2004). Baz may regulate adherens junction placement either directly, as it binds to β-catenin (Wei *et al*., 2005), or indirectly, e.g. through interactions with the actomyosin cytoskeleton or microtubules (Bertet *et al*., 2004; Zallen and Wieschaus, 2004; Harris and Peifer, 2007; Simões *et al*., 2010). Interestingly, Baz function as a modulator of adherens junction localization seems to be well conserved: in *C. elegans* epithelial cells, PAR-3 also mediates the initial clustering and apical localization of E-cadherin (Achilleos *et al*., 2010).

In mouse embryos, E-cadherin also plays an important role in the interplay between cell-to-cell communication and polarity. It becomes enriched in a cleavage post-compacted embryo in the contact cell surfaces (Vestweber *et al*., 1987). When it is depleted, aPKC is no longer restricted to apical surface in outer cells, but appears in all surfaces of these cells (Stephenson *et al*., 2010). This suggests that like PAC-1 in *C. elegans*, E-cadherin is necessary to establish PAR asymmetry in mouse. However, since depletion of E-cadherin also hinders cell-to-cell adhesion, it is difficult to interpret the molecular role of E-cadherin unambiguously. E-cadherin could possibly recruit other factors to cell contact regions that participate in the establishment of polarity, or it may be required for cells to sustain sufficient contacts with one another, thus priming a cadherin-independent mechanism.

**Figure 4:**
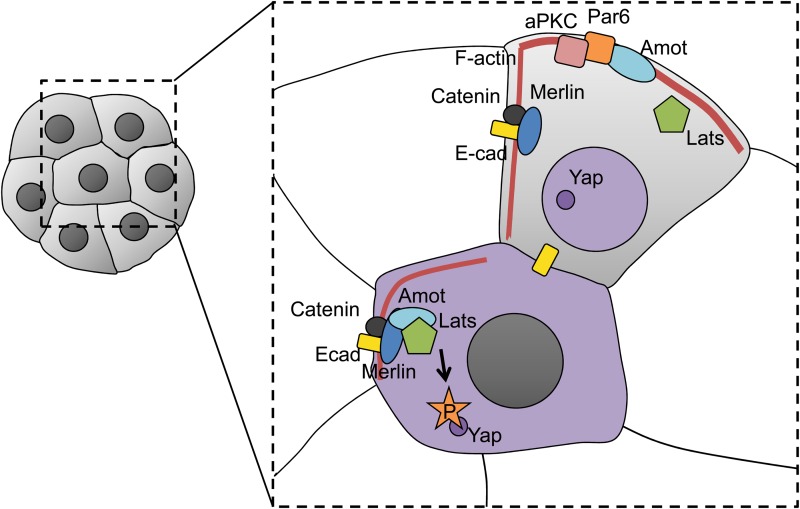
Regulation of Hippo pathway in 16-cell stage mouse embryo. Regulation of Yap phosphorylation and nuclear translocation in outer and inner cells. In outer cells, due to a lack of interaction between E-cadherin (E-cad), Merlin, Amot and Lats kinases Yap remains unphosphorylated and can access the nucleus, where it facilitates Cdx2 transcription. In inner cells, interaction between E-cad, Merlin and Amot activates Lats, leading to Yap phosphorylation and its sequestration in the cytoplasm. Details in the main text.

It has been suggested that E-cadherin may co-operate with the Hippo pathway (Hirate *et al*., 2013; Anani *et al*, 2014). The Hippo pathway is crucial for the regulation of organ growth and as such, it seems to be well conserved among species, from *C. elegans* to mammals (reviewed in Halder and Johnson, 2011; Yang and Hata, 2013). However, until now, it has only been shown to function during cleavage in mammalian embryos, where it is responsible for translating intercellular interactions into different cell fates in inner and outer cell populations (Nishioka *et al*., 2009; Stephenson *et al*., 2010; Hirate *et al*., 2013). The Hippo cascade regulates nuclear localization of transcriptional co-activator Yap and in consequence—the expression of Cdx2, a key determinant for differentiation into the trophectoderm lineage. In inner cells, where Hippo signalling occurs, Yap is phosphorylated by Lats protein kinase and localizes to the cytoplasm; in outer cells, where the pathway is inactive, Yap localizes to the nucleus, and regulate target genes promoting the trophoblast cell fate (Fig. 4) (Yagi *et al*., 2007; Nishioka *et al*., 2008, 2009; Wicklow *et al*., 2014). E-cadherin is required to exclude Yap from the nucleus of some inner cells (Nishioka *et al*., 2009; Stephenson *et al*., 2010), indicating that it may be needed for Hippo signalling, although it is difficult to discount an indirect requirement for proper cellular adhesion, which also depends on E-cadherin. The association between the cortical protein Angiomontin (Amot), known to activate the Hippo pathway (Hirate *et al*., 2013; Leung and Zernicka-Goetz, 2013), and E-cadherin may be especially important for asymmetric Hippo signalling (Hirate *et al*., 2013). In outer cells, Amot is localized at the apical, contact-free surfaces, whereas in inner cells, it is phosphorylated and enriched throughout the cell cortex. Amot binds to actin and E-cadherin, and this interaction is promoted by Nf2/Merlin (Cockburn *et al*., 2013; Hirate *et al*., 2013). It seems that a large protein complex of E-cadherin/α- and β-catenin/Merlin/Amot is formed at the contact sites and probably activates Lats kinase responsible for Yap phosphorylation (Gladden *et al*., 2010; Yi *et al*., 2011a, b; Hirate *et al*., 2013). Importantly, Amot colocalizes with E-cadherin only within inner cells, where its function is required, as it is excluded from basolateral surfaces in outer cells (Hirate *et al*., 2013; Leung and Zernicka-Goetz, 2013).

The asymmetric localization of Amot, as well as inner–outer differences in Hippo cascade activity, is determined by the blastomere polarity (Fig. 4). Without Par6 or aPKC, Hippo signalling is active and Yap localizes to the cytoplasm in both inner and outer cells (Hirate *et al*., 2013). This effect depends on cell adhesion, as dissociated cells lacking Par6 have nuclear Yap, like wild-type dissociated cells. Therefore, both cell polarity and cell adhesion are required for asymmetric Hippo signalling. Interestingly, some cells originating from asymmetric divisions can transiently localize to outer surfaces, even though they appear apolar (Anani *et al*., 2014). These cells are eventually internalized but can activate Hippo signalling while still on the surface of the embryo, suggesting that cell polarity rather than position regulates Hippo activity. The loss of Par6 or aPKC activity enables Amot to locate in the basolateral cortex in outer cells and therefore to colocalize with E-cadherin (Hirate *et al*., 2013). It seems likely that restriction of Amot only to the contact-free surfaces in outer cells, through the direct or indirect action of PAR proteins, prevents Amot from associating with E-cadherin at the contact sites, and in consequence inhibits Hippo signalling. Amot sequestration to the apical region in outer cells can be also mediated by actin-binding: Amot can bind actin, which is enriched in the apical cortex of outer blastomeres (Hirate *et al*., 2013). Additionally, actomyosin may affect the Hippo signalling via Rho and Rock GTPases. Their inhibition not only disrupts Par, Scribble, Lgl-1 and F-actin distribution in blastomeres, but also diminishes Yap nuclear localization in outer cells (Clayton *et al*., 1999; Liu *et al*., 2013; Kono *et al*., 2014).

Importantly, asymmetry in Cdx2 between outer and inner cells is also regulated by the asymmetric localization and inheritance of Cdx2 transcripts. During compaction, Cdx2 transcripts localizes to the apical domain of blastomeres, mirroring asymmetric localization of developmentally important transcripts in other non-mammalian vertebrate and invertebrate embryos. This localization of Cdx2 transcripts contributes to a process whereby Cdx2 expression becomes restricted to outer cells, thus biasing those cells to become trophectoderm (Skamagki *et al*., 2013). How many other transcripts might become asymmetrically localized and inherited to guide/bias cell fate remains still to be discovered.

## Human perspective

As we have shown in the previous paragraphs, Par-related mechanisms regulating the pattern of cleavage divisions are quite conserved among different phyla. Therefore, although there are no direct data on the mechanisms involved in polarity establishment or division plane orientation during cleavage in human embryos, it is highly likely that they are govern by interactions between the cytoskeleton, Par signalling and cell-to-cell communication analogical to those reported in other species. Consequently, a reliable assessment of these parameters may be very beneficial for selection of the highest quality embryos in IVF clinics.

How could this be achieved? One promising approach involves transcriptomic analysis of the embryonic material. Single-cell transcriptome analysis (Guo *et al*., 2010; Tang *et al*., 2010) enables development of feasible embryo selection protocols based on RNA analysis. Although at present, there are still not enough data correlating gene expression patterns in oocytes and blastomeres with embryonic developmental potential, the collection of transcriptome information in humans and animal models is in progress (Hamatani *et al*., 2004; Wang *et al*., 2004; Zhang *et al*., 2009; Robert *et al*., 2011; Vassena *et al*., 2011; Kakourou *et al*., 2013). Thus, these data will provide a necessary starting point for the design of appropriate transcriptome-based oocyte/embryo selection procedures. It is very plausible that assessment of the whole transcriptome will not be necessary to distinguish the most viable embryos, and that selected quality markers alone may be sufficient. RNAs of proteins involved in the establishment of polarization, cell-to cell communication and division planes are promising candidates for such biomarkers. Material for the RNA analysis can be obtained either from a biopsy of an oocyte's first polar body or a single blastomeres at 8-cell stage. It has been shown that transcriptome analysis of the polar body reflects well the transcriptome of the oocyte and is harmless for the future embryo (Reich *et al*., 2011). Since embryonic genome is activated in humans at 4- to 8-cell stage (Dobson *et al*., 2004; Zhang *et al*., 2009), maternal transcripts regulate at least first three rounds of cleavage divisions and therefore their analysis might provide important information about developmental potential of the embryo. Eight-cell stage blastomeres, which can be an alternative source of RNA, are biopsied routinely in preimplantation genetic diagnosis (PGD) and screening (PGS) and it has been proved that embryos devoid of a single 8-cell stage blastomere develop to term normally (Harper *et al*., 2010; Harton *et al*., 2011; Ajduk and Zernicka-Goetz, 2013).

An alternative approach to assess blastomere polarization and mechanism of cleavage orientation involves time-lapse imaging. Analysis of recordings obtained from automated visualization systems allows the identification of cleavage patterns at 2- to 4-cell stage transition and therefore the distinction of ME, EM, MM and EE embryos. As shown in mouse, such embryos have clearly different developmental potentials (Piotrowska-Nitsche and Zernicka-Goetz, 2005; Piotrowska-Nitsche *et al*., 2005). Importantly, this observation has been confirmed in humans: embryos with planar morphology (corresponding probably to EE and MM embryos) have significantly reduced rates of blastocyst formation and implantation (Ebner *et al*., 2012). Time-lapse imaging also helps to assess the exact timing of embryo compaction (a visual sign of blastomere polarization and adhesion). This morphokinetic parameter has been correlated with embryonic developmental potential, particularly the ploidy status: euploid embryos required a significantly shorter time to initiate compaction than aneuploid ones (Campbell *et al*., 2013). Interestingly, it has also been shown that diagnostic blastomere biopsy (PGD) conducted at the 6- to 8-cell stage delays compaction, indicating that PGD may interfere with embryo polarization and formation of potentially important cell-to-cell contacts (Kirkegaard *et al*., 2012). However, it remains to be elucidated how delayed polarization caused by the blastomere biopsy may affect further embryonic development, e.g. subsequent embryonic cell differentiation.

Time-lapse imaging also enables functional assessment of another key polarization-related factor, the actomyosin cytoskeleton. Frequent visualization (every 10 s) of mouse zygotic cytoplasm in a period directly after fertilization has identified rhythmic cytoplasmic movements dependent on actin and myosin interactions (Ajduk *et al*., 2011). Embryos with slow cytoplasmic movement, reflecting poor quality of the actomyosin network, showed lower developmental potential as assessed by the developmental stage and cell number reached after 4 days in culture and their ability to develop to term (Ajduk *et al*., 2011). Such cytoplasmic flows have also been described in human 1-cell embryos (Swann *et al*., 2012), although there are still no data correlating cytoplasmic dynamics with developmental quality of the human embryos.

In summary, mechanisms regulating blastomere polarization and orientation of division plane are highly conserved among distinct phyla, indicating that these developmental features are crucial for proper embryonic development. Therefore, it is worth considering whether their assessment, based either on visual cues provided by time-lapse imaging or on molecular analysis, could be incorporated into embryo selection protocols as an additional factor describing embryonic quality.

## Author's roles

A.A. and M.Z.-G. prepared the manuscript.

## Funding

A.A. is a beneficent of the National Science Centre grant (UMO-2012/07/D/NZ5/04301). M.Z.-G. thanks the Wellcome Trust for supporting the work in her laboratory. Funding to pay the Open Access publication charges for this article was provided by the Wellcome Trust.

## Conflict of interest

The authors do not have any conflict of interest.
